# Changes in rates of psychiatric beds and prison populations in sub-Saharan Africa from 1990 to 2020

**DOI:** 10.7189/jogh.12.04054

**Published:** 2022-09-03

**Authors:** Adrian P Mundt, Sabine Delhey Langerfeldt, J Maphisa Maphisa, Oumar Sourabié, Blaise Nguendo Yongsi, Enzo Rozas Serri, Jean C Bukasa Tshilonda, Jeronimo H Te, Mary A Bitta, Lipalesa Mathe, Olive Liwimbi, Palmira Fortunato dos Santos, Olayinka Atilola, Stefan Jansen, Jean A Diegane Tine, Clementina Akran, Abdul Jalloh, Ashraf Kagee, Elizabeth S Van Wyk, Jimmy B Forry, Mwiya Liamunga Imasiku, Handrick Chigiji, Stefan Priebe

**Affiliations:** 1 Medical Faculty, Universidad Diego Portales, Santiago, Chile; 2 Departamento de Neurología y Psiquiatría, Clínica Alemana de Santiago, Facultad de Medicina Clínica Alemana, Universidad del Desarrollo, Santiago, Chile; 3 Department of Psychology, University of Botswana, Gaborone, Botswana; 4 Psychiatrist, Regional Center Hospital of Fada N’gourma, Fada N’gourma, Burkina Faso; 5 Institute for Training & Research in Population Studies, University of Yaoundé, Yaoundé, Cameroon; 6 Department of Psychiatry and Mental Health, Hospital Clínico Universidad de Chile, Santiago, Chile; 7 Higher Institute of Medical Techniques Mbujimayi, Mbujimayi, Democratic Republic of Congo; 8 West African Epidemiology Network on Drug Use (WENDU), Bissau, Guinea-Bissau; 9 Department of Psychiatry, University of Oxford, Oxford, United Kingdom; 10 KEMRI-Welcome Trust Research Program, Kilifi, Kenya; 11 The National University of Lesotho, Maseru, Lesotho; 12 Zomba Mental Hospital, Ministry of Health, Zomba, Malawi; 13 Ministry of Health, Mental Health Department, Maputo, Mozambique; 14 Lagos State University College of Medicine (LASUCOM), Lagos, Nigeria; 15 College of Medicine and Health Sciences, University of Rwanda, Kigali, Rwanda; 16 Institute of Health and Development, Cheikh Anta Diop University, Dakar, Senegal; 17 Statistics Sierra Leone, Tower Hill, Freetown, Sierra Leone; 18 Sierra Leone Psychiatric Teaching Hospital, Freetown, Sierra Leone; 19 College of Medicine and Allied Health Sciences, University of Sierra Leone, Freetown, Sierra Leone; 20 Alan Fisher Centre for Public Mental Health, University of Cape Town, Cape Town,; South Africa; 21 Department of Psychology, Stellenbosch University, Stellenbosch, South Africa; 22 Department of Psychiatry, Faculty of Medicine, Mbarara University of Science and Technology, Mbarara, Uganda; 23 Department of Mental Health and Psychiatry, Faculty of Clinical Medicine and Dentistry, Kampala, International University-Western Campus, Bushenyi, Uganda; 24 Department of Psychiatry, Mubende Regional Referral Hospital, Mubende, Uganda; 25 Department of Psychiatry, University of Zambia, Lusaka, Zambia; 26 Zimbabwe National Statistics Agency, Harare, Zimbabwe; 27 Unit for Social and Community Psychiatry, Queen Mary University of London, London, UK

## Abstract

**Background:**

Psychiatric bed numbers (general, forensic, and residential) and prison populations have been considered indicators of institutionalization. The present study aimed to assess changes of those indicators across sub-Saharan Africa (SSA) from 1990 to 2020.

**Methods:**

We retrospectively obtained data on psychiatric bed numbers and prison populations from 46 countries in SSA between 1990 and 2020. Mean and median rates, as well as percentage changes between first and last data points were calculated for all of SSA and for groups of countries based on income levels.

**Results:**

Primary data were retrieved from 17 out of 48 countries. Data from secondary sources were used for 29 countries. From two countries, data were unavailable. The median rate of psychiatric beds decreased from 3.0 to 2.2 per 100 000 population (median percentage change = -16.1%) between 1990 and 2020. Beds in forensic and residential facilities were nonexistent in most countries of SSA in 2020, and no trend for building those capacities was detected. The median prison population rate also decreased from 77.8 to 71.0 per 100 000 population (-7.8%). There were lower rates of psychiatric beds and prison populations in low-income and lower-middle income countries compared with upper-middle income countries.

**Conclusions:**

SSA countries showed, on average, a reduction of psychiatric bed rates from already very low levels, which may correspond to a crisis in acute psychiatric care. Psychiatric bed rates were, on average, about one twenty-fifth of countries in the Organization for Economic Co-operation and Development (OECD), while prison population rates were similar. The heterogeneity of trends among SSA countries over the last three decades indicates that developments in the region may not have been based on coordinated policies and reflects unique circumstances faced by the individual countries.

Sub-Saharan Africa (SSA) comprises 48 countries with significant political, historical, social, and economic commonalities, but also important differences. Mental health services are limited in the region; in particular, the number of psychiatric beds is low [1]. Mental health facilities in SSA are usually centralized in metropolitan areas, which often leaves people residing in rural areas with less access. In addition, many SSA countries still lack mental health legislation and policies to protect fundamental rights, with the lowest rate of stand-alone mental health law worldwide (44%) [2]. Mental and substance use disorders account for 19% of the burden of disease in the region, which is close to the worldwide burden of 23% [3].

In prison populations, mental health and substance use disorders are highly prevalent globally [4-8], and remain untreated, underestimated and undetected, especially in low-income and middle-income countries (LMICs) [5,9,10]. African prisons are often characterized by lack of resources, overcrowding, deficient infrastructure, food scarcity, and lack of safety, conditions which can aggravate mental illnesses [11-13]. Additionally, infectious diseases, especially HIV and tuberculosis, are a major health concern in SSA prisons that can interact with mental health problems in this population [14].

Western-style mental health care systems, including psychiatric hospitals, were mostly built in SSA during colonial times starting in the 19th century [15,16]. Policies regarding mental health care reforms have also been implemented with varying degrees of success [17,18]. A recent systematic review of expert arguments for changes of psychiatric bed rates reported few recommendations from LMICs and a majority of those arguing to increase rates [19]. The development of community-based public health services and primary health care has often been limited by a lack of resources [20], and some countries in SSA have not yet made transitions to community based mental health care systems. In some places, community care systems are not yet sufficiently equipped. Several SSA countries that have been able to fund hospitals have been described to have hospital-based systems, while others may have hardly any services available [21].

Several Western high-income countries and also middle-income regions have undergone substantial psychiatric bed removals in past decades [19,22,23], while expanding imprisonment. In Latin America, psychiatric bed removals and the increase of prison population rates were associated [23]. Access to mental-health services, rate of involvement in crime, and subsequent incarceration are all inextricably linked. This is evidenced from contemporary research, from high-income countries, which have observed that there is an increased risk of involvement in crime after onset of mental disorder [24] and that access to mental health services can and does reduce rates of criminalization [25]. Based on these observations, the initiatives to improve the efficiency of either of the two sectors should be integrated. Some high-income countries such as the United States of America and The Netherlands have evolved integrated mental health and criminal justice initiatives to jointly address the relationship between mental disorders and crime [26,27]. Developments in SSA towards this direction should be informed by data. Thus, the aim of the present study was to assess rates and trends of availability of psychiatric beds (a proxy for access to mental health services in persons suffering from severe mental illness) and the prison population across SSA countries in the last three decades (1990 and 2020).

## METHODS

### Data Sources

We formed an international network of researchers from SSA between April 2019 and December 2020 and conducted a retrospective database study. Collaborators were contacted based on their participation as authors in scientific journals, authorship of the World Health Organization (WHO)-AIMS reports, personal networks, and snowballing. We also contacted ministries of health and related government institutions. We asked potential collaborators who could not participate if they knew somebody in the country or in the surrounding countries who may be willing to participate. Communication in the research network was via email in the English language. A template was used to collect national data from each year between 1990 until 2020. Data collection took place between April 2019 and December 2020. Researchers from 13 countries participated in the network: Botswana, Burkina Faso, Cameroon, Democratic Republic of Congo, Guinea-Bissau, Kenya, Malawi, Mozambique, Rwanda, Senegal, Sierra Leone, Uganda, and Zambia. When data from primary or secondary national sources were unavailable, prison population rates were retrieved from the World Prison Brief online database [28] and psychiatric bed counts from WHO between 1990 and 2020 [29]. Somalia and Sudan were excluded from the data analysis, as data on psychiatric bed numbers were not reported by the WHO for these countries. Psychiatric bed numbers for Organization for Economic Co-operation and Development (OECD) countries were retrieved from www.stats.oecd.org [30]. Prison population rates of the OECD countries were retrieved from the Institute of Criminal Policy Research (www.prisonstudies.org) [28].

### Definition of indicators

Rates were calculated as the number of psychiatric beds and prisoners per 100 000 population based on population estimates provided by the World Bank [31]. For Eritrea, total population numbers between 2015 and 2020 were retrieved from the World Population Review [32], as these were unavailable in the World Bank database. Four different indicators were assessed: 1) Psychiatric beds, which were defined as all beds in hospital settings provided to treat people with mental health problems in psychiatric hospitals or in psychiatric units in general hospitals, including beds specifically assigned for children and adolescents. Private psychiatric beds were excluded when separately reported. 2) Forensic psychiatric beds, which included any bed assigned for the assessment or treatment in forensic psychiatry ordered by law or courts. If bed numbers for forensic psychiatric care and child and adolescent psychiatric care were specified as separate from general psychiatry, they were also added to the numbers of psychiatric beds. In many LMICs, such beds are not separately specified but general psychiatric beds are flexibly used for such purpose. 3) Beds in residential or housing facilities for people with mental disorders, which included community-based mental health care facilities that provide overnight residence, mostly serving patients with stable mental illnesses and patients that do not require acute medical treatment. Facilities specifically offering treatment for people with substance use disorders or intellectual disability were excluded, as well as any generic facility not specifically intended for mental health care needs (eg, rest and nursing homes for elderly people). 4) Prisoners were defined as all people in fulltime incarceration in jails or prisons. We excluded people on probation, parole, or serving alternative sentences that imply only daytime or nighttime in prison.

### Statistical analysis

We calculated the percentage changes in rates of psychiatric beds and prison population rates between the first and last available data points, and the median and mean values with interquartile ranges and standard deviation. The median and mean values for percentage changes were calculated in order to present a descriptive analysis of the percentage changes. The Shapiro-Wilk test was used to test for normal distribution. Changes in absolute numbers of psychiatric beds and prison populations were also calculated for all countries to estimate absolute changes in the region since 1990. In addition, we compared median and mean values for a priori defined groups according to income level at the last data point, and calculated percentage changes for the median and mean values over this timeframe. These findings were presented as descriptive analysis. We compared findings in SSA with countries that form part of the OECD, an international organization of 37 countries, most of which are high-income.

We did not involve patients and the public in the study design. Research findings will be disseminated to the WHO regional offices and user organizations in Africa.

## RESULTS

Primary data on rates of psychiatric beds and prison populations were retrieved from 17 out of 48 countries in SSA (Botswana [33], Burkina Faso, Cameroon [34-36], Democratic Republic of Congo [37], Guinea-Bissau, Kenya, Lesotho, Malawi, Mozambique [38,39], Nigeria, Rwanda, Senegal, Sierra Leone, South Africa, Uganda, Zambia, and Zimbabwe). In four countries (Lesotho, Nigeria, South Africa and Zimbabwe), primary data was available only for the prison populations (**Table 1**). In 28 countries, we had contact with at least one collaborator who either could not obtain the data or did not sustain the contact, including South Sudan, from where it was reported that the data registry was destroyed in the war. For these countries, data were included from secondary sources. The total population of SSA in 2020 amounted to more than 1160 million people. Primary data on specialized forensic psychiatric beds were available in 11 countries, and collaborators from nine countries provided data on residential facilities.

**Table 1:** Rates of psychiatric beds, specialized forensic psychiatric beds, places in residential facilities for individuals with mental health problems, and prison populations in 46 sub-Saharan African countries

	Psychiatric beds per 100 000 population				Specialized forensic psychiatric beds per 100 000 population				Beds in residential facilities per 100 000 population				Prison population per 100 000 population			
**Country**	**Period of observation**	**First data point**	**Last data point**	**Percentage change**	**Period of observation**	**Rate at first point**	**Rate at last point**	**Percentage change**	**Period of observation**	**Rate at first point**	**Rate at last point**	**Percentage change**	**Period of observation**	**Rate at first point**	**Rate at last point**	**Percentage change**
**Angola**	2001-2017	1.3	0.8	-40.0	2017	ND	0.1	NA	2017	ND	0.3	NA	1999-2016	32.4	83.2	156.5
**Benin**	2001-2020	0.3	4.7	1453.3	NA	ND	ND	NA	2011-2020	5.4	6.8	24.9	1997-2020	60.1	74.2	23.4
**Botswana**	1990-2020	11.0	16.4	48.2	NA	ND	ND	NA	1990-2019	0.0	0.0	0.0	1992-2019	198.2	229.5	15.8
**Burkina Faso**	1990-2020	1.7	0.9	-50.6	1990-2020	0.0	0.0	0.0	1990-2020	0.1	0.2	68.9	1995-2020	16.4	37.0	125.9
**Burundi**	2001-2005	1.0	1.0	0.0	2017	ND	0.2	NA	NA	ND	ND	NA	1996-2020	124.2	96.0	-22.7
**Cabo Verde**	2001-2011	7.8	9.4	20.0	NA	ND	ND	NA	NA	ND	ND	NA	1997-2018	154.4	296.0	91.8
**Cameroon**	1991-2019	1.4	0.3	-79.7	NA	ND	ND	NA	1990-2011	0.2	0.1	-47.6	1990-2020	134.2	85.0	-36.7
**Central African Republic**	2001-2014	0.7	1.4	98.6	NA	ND	ND	NA	NA	ND	ND	NA	1995-2020	31.0	16.0	-48.4
**Chad**	2001-2011	0.2	0.02	-90.0	2017	ND	0.01	NA	NA	ND	ND	NA	1994-2020	42.3	59.0	39.5
**Comoros**	2001-2011	0.0	0.4	NA	NA	ND	ND	NA	NA	ND	ND	NA	1998-2020	38.8	37.0	-4.7
**Republic of Congo**	2001-2014	1.5	2.0	31.3	NA	ND	ND	NA	NA	ND	ND	NA	1993-2019	35.9	27.0	-24.7
**Côte d'Ivoire**	2001-2014	1.5	1.3	-16.7	NA	ND	ND	NA	NA	ND	ND	NA	1993-2020	92.0	82.0	-10.9
**Democratic Republic of Congo (DRC)**	1990-2019	0.4	0.6	65.7	1990-2019	0.0	0.0	0.0	1990-2020	0.0	0.3	621.1	1990-2019	612.5	48.2	-92.1
**Eritrea**	2001-2020	6.4	5.9	-8.0	NA	ND	ND	NA	2020	ND	3.4	NA	NA	ND	ND	NA
**Equatorial Guinea**	2001-2005	0.0	0.0	0.0	NA	ND	ND	NA	NA	ND	ND	NA	2015	ND	42.8	NA
**Eswatini**	2011-2014	12.5	15.2	21.4	NA	ND	ND	NA	NA	ND	ND	NA	1997-2020	229.7	277.0	20.6
**Ethiopia**	2001-2014	0.7	0.4	-47.1	NA	ND	ND	NA	2020	ND	0.2	NA	1990-2020	55.4	99.0	78.7
**Gabon**	2001-2011	6.0	8.0	33.2	NA	ND	ND	NA	NA	ND	ND	NA	2006-2018	192.3	241.0	25.3
**The Gambia**	2001-2014	7.8	5.2	-32.8	NA	ND	ND	NA	2020	ND	2.6	ND	1999-2019	37.4	31.0	-17.2
**Ghana**	2001-2020	10.1	6.3	-38.1	NA	ND	ND	NA	2011-2020	0.1	0.3	230.0	1995-2018	45.4	50.0	10.1
**Guinea**	2001-2014	0.5	1.3	156.0	NA	ND	ND	NA	2014	ND	0.2	NA	1996-2018	45.4	28.0	-38.4
**Guinea-Bissau**	1990-1998	6.2	5.2	-16.1	1990-2019	0.0	0.0	0.0	1990-2019	0.0	0.0	0.0	2017	10.7	10.7	0.0
**Kenya**	2001-2017	3.5	2.5	-27.4	2000-2019	0.0	0.0	0.0	2000-2019	0.0	0.0	0.0	1992-2020	114.2	81.0	-29.1
**Lesotho**	2001-2014	11.0	11.7	6.6	2019	ND	1.7	NA	NA	ND	ND	NA	1990-2014	128.5	86.3	-32.8
**Liberia**	2017-2020	1.9	1.9	-2.6	NA	ND	ND	NA	2017	NA	0.4	NA	2007-2019	29.5	54.7	85.2
**Madagascar**	2001-2017	1.6	1.3	-17.5	NA	ND	ND	NA	2017-2020	0.1	0.1	0.0	1990-2020	178.8	99.0	-44.6
**Malawi**	1990-2020	4.4	2.4	-44.8	1990-2020	0.0	0.0	0.0	1990-2020	0.0	0.0	0.0	1990-2020	72.0	71.0	-1.4
**Mali**	2001-2014	1.0	2.0	95.0	NA	ND	ND	NA	NA	ND	ND	NA	1995-2020	45.7	34.0	-25.7
**Mauritania**	2001-2005	2.0	2.0	0.0	NA	ND	ND	NA	NA	ND	ND	NA	1997-2018	57.5	52.7	-8.4
**Mauritius**	2001-2020	80.0	51.1	-36.1	NA	ND	ND	NA	2011	ND	0.0	NA	1990-2018	91.8	194.0	111.3
**Mozambique**	2001-2019	2.4	1.8	-25.0	1990-2019	0.0	0.0	0.0	1990-2019	0.0	0.0	0.0	1996-2020	44.6	57.0	27.8
**Namibia**	2001-2014	15.0	12.0	-19.9	2011	ND	3.3	NA	2011-2017	0.0	0.5	NA	1992-2016	178.7	319.0	78.5
**Niger**	2001-2014	2.0	1.8	-11.5	NA	ND	ND	NA	NA	ND	ND	NA	1998-2019	49.9	40.0	-19.9
**Nigeria**	2001-2014	3.4	1.5	-55.0	NA	ND	ND	NA	NA	ND	ND	NA	1990-2020	56.8	31.0	-45.3
**Rwanda**	1990-2020	1.9	4.1	56.8	1990-2019	0.0	0.0	0.0	1990-2019	0.2	0.2	0.0	1998-2020	1861.0	545.0	-70.7
**São Tomé and Príncipe**	2001-2011	21.0	36.3	72.8	NA	ND	ND	NA	NA	ND	ND	NA	1990-2018	76.3	116.0	52.0
**Senegal**	2001-2020	2.5	1.5	-20.0	2017	ND	0.1	NA	2011-2020	0.5	0.9	72.5	1994-2019	47.7	68.0	42.6
**Seychelles**	2001-2020	89.0	60.4	-21.7	NA	ND	ND	NA	NA	ND	ND	NA	2000-2020	204.6	322.0	57.4
**Sierra Leone**	2000-2019	4.4	3.8	-13.6	1990-2019	0.0	0.0	0.0	2001-2014	0.03	0.0	NA	2004-2020	25.8	47.0	82.5
**South Africa**	2001-2020	43.8	33.7	-23.0	NA	ND	ND	NA	2011	ND	3.5	NA	199-2020	298.8	211.4	-29.3
**South Sudan**	2014-2017	0.1	0.1	-28.6	NA	ND	ND	NA	2014	ND	0.0	NA	2019	ND	50.0	NA
**Tanzania**	2001-2020	4.0	2.3	-41.5	NA	ND	ND	NA	2011-2020	0.8	0.1	-87.5	1995-2020	141,3	52.0	-63.2
**Togo**	2001-2017	4.0	2.0	-49.5	2017	ND	0.5	NA	2011-2017	0.0	0.3	NA	1994-2020	79.3	50.0	-37.0
**Uganda**	2001-220	3.4	3.7	8.2	2005-2019	3.4	0.3	-92.4	NA	ND	ND	NA	1993-2020	99.5	142.0	42.7
**Zambia**	1990-2020	6.2	2.8	-54.8	1990-2019	0.4	0.2	-50.0	2011-2017	0.5	0.3	-37.8	1998-2019	145.1	123.0	-15.2
**Zimbabwe**	2001-2017	11.0	12.5	13.7	2017	ND	1.4	NA	2011-2017	1.5	0.5	-65.3	1990-2017	152.1	114.0	-25.1
																
**MEDIAN**		3.0	2.2	-16.1		0.0	0.0	0.0		0.1	0.2	0.0		77.8	71.0	-7.8
**IQR**		6.1	4.9	58.1		0.0	0.2	0.0		0.3	0.5	45.3		105.0	69.0	73.7
**MEAN**		8.7	7.4	28.8		0.4	0.4	-15.8		0.5	0.8	48.7		151.4	109.9	9.9
**SD**		18.0	12.9	223.1		1.1	0.9	33.1		1.3	1.5	168.7		289.3	107.0	56.8
**Absolute numbers (thousands)**		38	39	4.1		0.2	0.7	358.8		1.3	3.9	112.3		789	853	8.2
**Total population (millions)**		619	989	59.6		146	392	168.2		248	676	172.3		508	1061	108.9
**Number of countries**		46	46	45		10	18	9		19	28	16		42	45	42

NA – not applicable, ND – no data, IQR – interquartile range, SD – standard deviation

Most countries included in this study were low-income in 1992, except for eight countries that were lower-middle income countries (Angola, Cabo Verde, Cameroon, Republic of Congo, Côte d'Ivoire, Eswatini, Namibia, and Senegal) and five that were upper–middle income at the first data point (Botswana, Gabon, Mauritius, Seychelles, and South Africa). South Sudan fulfilled criteria for a low-income country in 2020. The income group of 15 countries increased by 2020, and all other countries remained in the same income group over the period of observation. Mauritius and Seychelles were the only countries that became high-income during the timeframe. Since the data showed a significant deviation from normality, median rates were preferred as measures of central tendency.

### Psychiatric Beds

**Figure 1:**
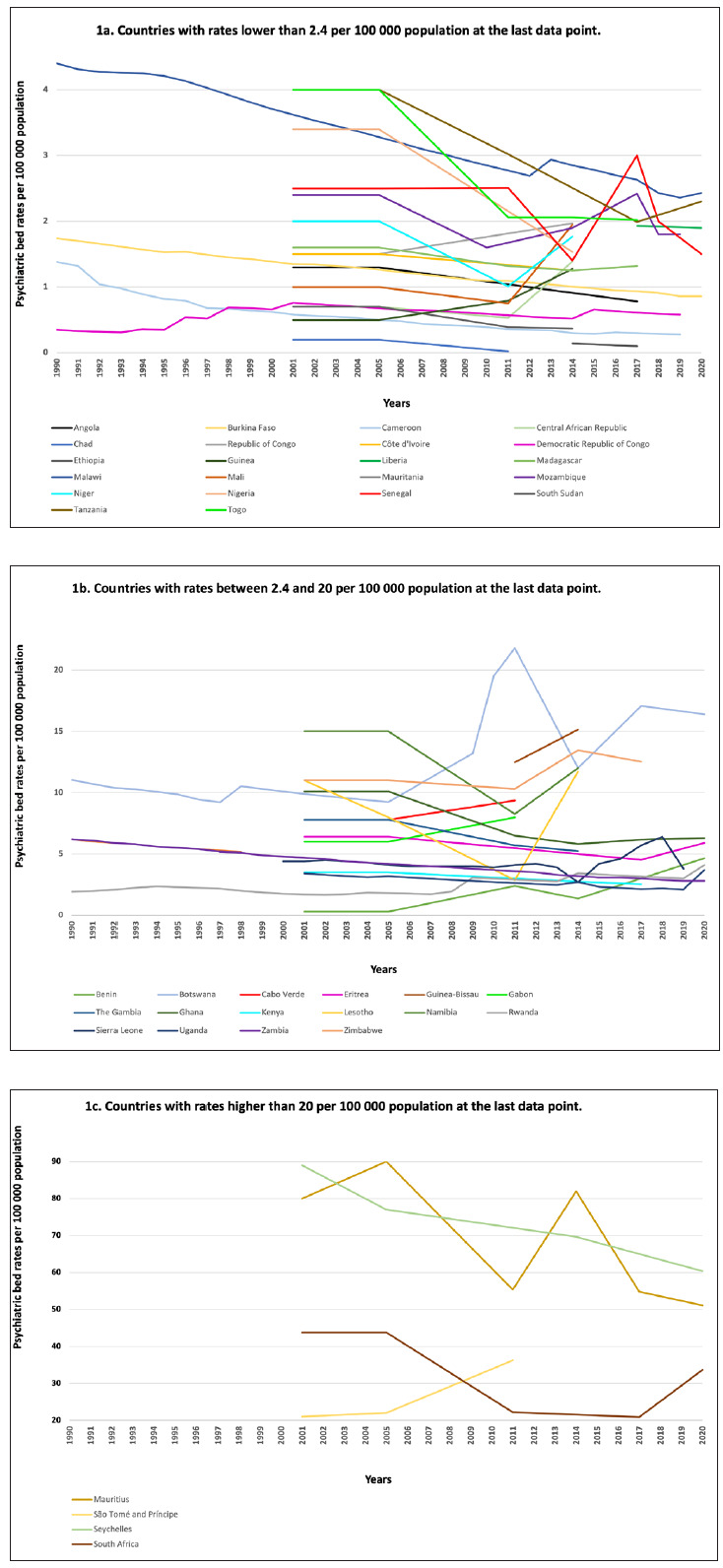
Rates of psychiatric beds per 100 000 population (1990-2020). **Panel A.** Countries with rates lower than 2.4 per 100 000 population at the last data point; **Panel B.** Countries with rates between 2.4 and 20 per 100 000 population at the last data point; **Panel C.** Countries with rates higher than 20 per 100 000 population at the last data point;

Median rates of psychiatric beds decreased from 3.0 to 2.2 per 100 000 population (median percentage change -16.1%; -0.8 per 100 000 population), ranging from the highest percentage increase in Benin (+1453.3%, +4.4 beds per 100 000) to the strongest percentage decrease in Chad (-90.0%, -0.2 per 100 000). Most countries (27 out of 46) showed decreased psychiatric bed rates over the period of observation, comparing the first to the last data point (**Figure 1****,** Panel A, Panel B, Panel C). Cameroon, [34-36] Chad, Equatorial Guinea, and South Sudan had the lowest psychiatric bed rates, all below 0.1 per 100 000 population at the last data point. For Equatorial Guinea, no psychiatric beds were reported over the entire timeframe. Seychelles had the highest rate in the region (60.4), followed by Mauritius (51.1), and São Tomé and Príncipe (36.3). For the entire region, the total number of psychiatric beds at the first data point was 37 579 and 39 137 at last data point, a difference of +4.1%.

### Specialized forensic psychiatric beds

Most countries (28 out of 46) did not have information on specialized forensic psychiatric beds. Out of 18 countries that had data on forensic psychiatric beds, eight countries reported not to have had any between 1990 and 2020 (Burkina Faso, Democratic Republic of Congo [37], Guinea-Bissau, Kenya, Malawi, Mozambique [38,39], Rwanda [40], and Sierra Leone; **Table 1**). In the remaining ten countries (Angola, Burundi, Chad, Lesotho, Namibia, Senegal, Togo, Uganda, Zambia, and Zimbabwe), rates ranged from 0.01 to 3.4 per 100 000 population. None of the countries reported an increase of forensic psychiatric beds. Uganda reported the strongest decrease of 92.4% (-3.1 beds per 100 000 population; see Figure S1 in the **Online Supplementary Document**). The total number of forensic psychiatric beds reported was 148 at the first data point and 679 at the last data point (+358.8%). Median rates of specialized forensic beds remained in 0.0 per 100 000 population during the period of observation, while the mean rates showed a slight increase from 0.38 to 0.43 per 100 000 population.

### Places in residential facilities

Rates of beds in residential facilities were available for 28 countries. Five countries (Botswana [33], Guinea-Bissau, Kenya, Malawi, and Mozambique [38,39]; **Table 1**) reported not to have residential facilities in the timeframe. Benin had the highest rate with 6.8 per 100 000 population at the last data point. Four countries presented a decrease and five countries an increase of the rates of residential facilities over time. Nine countries reported data for one year only and were not included in the trend analysis (Figure S2 in the **Online Supplementary Document**). Median rates of places in residential facilities increased from 0.1 to 0.2 per 100 000 from first to last data points and mean rates went up from 0.5 to 0.8 per 100 000 population. The total number of reported beds in residential facilities at the first data point was 1281, and 3912 at the last data point for the entire region (increase of 205.4%).

### Prison populations

**Figure 2:**
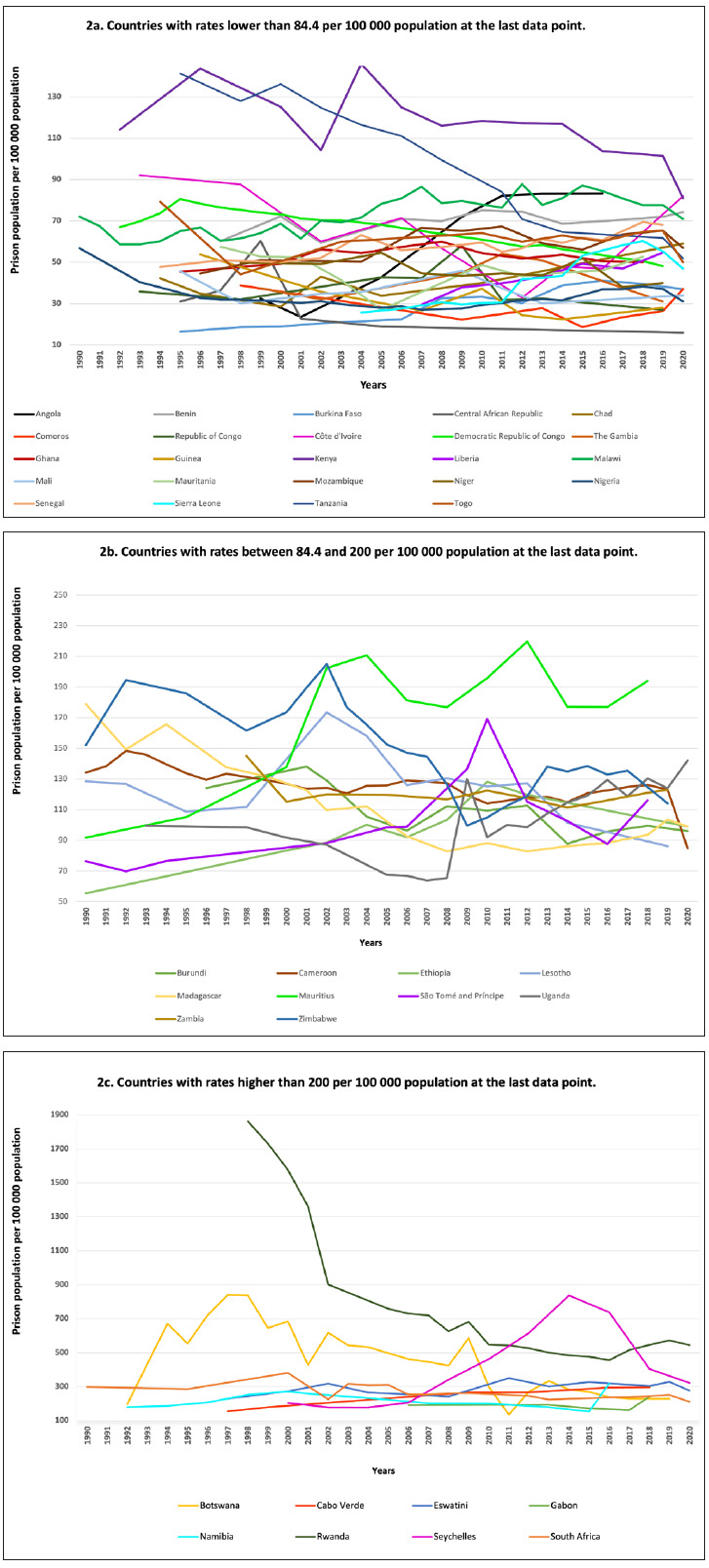
Rates of prison populations per 100 000 population (1990-2020). **Panel A.** Countries with rates lower than 84.4 per 100 000 population at the last data point; **Panel B.** Countries with rates between 84.4 and 200 per 100 000 population at the last data point; **Panel C.** Countries with rates higher than 200 per 100 000 population at the last data point.

Rates of prison populations were heterogeneous. They ranged from 10.7 per 100 000 population in Guinea-Bissau to 545 per 100 000 population in Rwanda at the last data point. Prison population rates increased in 19 countries and decreased in 23 countries, ranging from the highest increase of 156.5% in Angola (+50.8 per 100 000 population) to the strongest decrease of -92.1% in the Democratic Republic of Congo (-564.3 per 100 000 population). Median prison population rates decreased from 77.8 to 71.0 per 100 000 population, corresponding to a decrease of 7.8% over the period of observation (-6.8 per 100 000 population; **Figure 2****,** Panel A, Panel B, Panel C). The total number of imprisoned individuals reported in the region were 788 699 at the first data point, and 853 351 in 2020. It was not possible to assess trends for Equatorial Guinea, Guinea-Bissau and South Sudan, as prison population rates for these countries were only available for one year. Furthermore, there were no data available for Eritrea over the entire period of observation.

### Income groups

Low-income countries had the lowest levels of psychiatric beds and prison population rates and a small increase in both between 1990 and 2020. Lower-middle income countries had low levels of both indicators, but a decrease in both. Upper-middle income countries had median psychiatric bed rates six times greater than lower-middle income countries at the last data point and median prison population rates more than three times greater. Both indicators increased in upper-middle income countries over time. The only two high-income countries (Mauritius and Seychelles) had median psychiatric bed rates more than six times greater than upper-middle income countries at the last data point and also greater median prison population rates (**Table 2**). Specialized forensic psychiatric beds were typically unavailable in low-income and in lower–middle income economies. The rates of places in residential facilities for people with mental disorders were very low irrespective of the income group.

**Table 2:** Median rates of psychiatric beds, specialized forensic psychiatric beds, residential places, and prison population by income group in 2020

	Psychiatric beds per 100 000 population			Specialized forensic beds per 100 000 population			Beds in residential facilities per 100 000 population			Prison population per 100 000 population		
	**Rate at first point**	**Last data point**	**Percentage change**	**Rate at first point**	**Rate at last point**	**Percentage change**	**Rate at first point**	**Rate at last point**	**Percentage change**	**Rate at first point**	**Rate at last point**	**Percentage change**
**Low**	1.8	1.8	-1.6	0	0	0	0	0.2	1100.0	47.8	50.0	4.5
**Number of countries**	20	21		8	11		8	12		19	21	
**Lower-middle**	3.5	2.4	-29.3	0.2	0.1	-37.5	0.5	0.3	-31.3	84.2	81.5	-3.2
**Number of countries**	18	18		2	6		8	9		18	18	
**Upper-middle**	11.0	12.0	8.8	0	3.3	NA	0	0.5	NA	192.3	229.5	19.3
**Number of countries**	5	5		0	1		2	3		5	5	
**High**	84.5	70.2	-16.9	ND	ND	NA	ND	0	NA	148.2	258.0	74.1
**Number of countries**	2	2		0	0		0	1		2	2	

ND – no data reported, NA – not applicable

### Comparison with OECD countries

**Figure 3:**
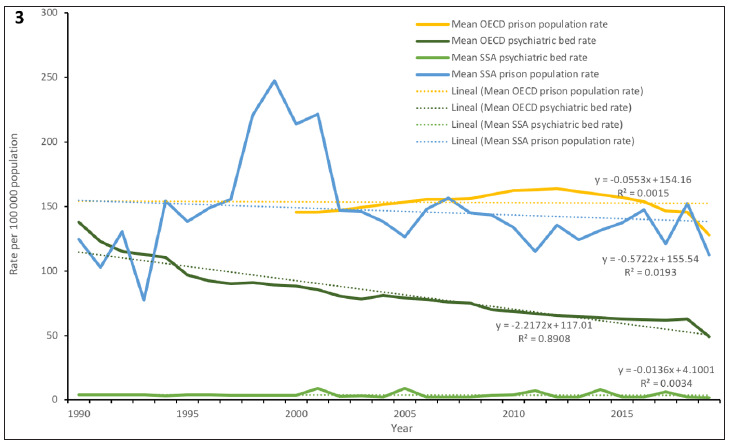
Mean psychiatric bed and prison population rates in sub-Saharan African countries compared with OECD countries (1990-2020)

In 2019, mean rates of psychiatric beds in SSA countries were on average about 25 times lower than in OECD countries (2.0 vs 49.3 per 100 000), while mean prison population rates were similar in the two groups of countries (112.5 in SSA vs 127.8 in OECD per 100 000). The mean psychiatric bed rates in SSA decreased between 1990 and 2020, which was in line with reductions in OECD countries. The mean prison population rates also decreased in both groups of countries (**Figure 3**).

## DISCUSSION

### Main findings

Rates of psychiatric beds decreased in a majority of SSA between 1990 and 2020 from initially already very low rates. Prison population rates showed heterogeneous trends from rates comparable to OECD countries. Both indicators showed a direct relationship with the income level of the country. Specialized forensic psychiatric beds and places in residential facilities for people with mental health illnesses were typically not specifically reported, unavailable or had negligible capacities in most countries.

### Interpretation

Many SSA countries underwent decreases in psychiatric bed rates, as in Western countries, but probably for very different reasons. One is faster population growth than in the rest of the world [31]. Median rates of psychiatric beds across SSA had already been the lowest among LMICs worldwide [23]. The mean rate of two beds per 100 000 population observed at the last data point was about one twenty-fifth of the rate typically seen in OECD countries and recommended in Western high-income countries [41] and may likely indicate a crisis for acute mental health care. The low number of psychiatric beds can correspond to lower mental health spending in poorer countries and health budgets with competing demands for infectious diseases that have largely been prioritized [42]. It is unclear how much of the resources saved from removals of psychiatric beds in SSA countries have actually been transferred to community-based and outpatient care. Furthermore, the capacity in facilities of poorly-resourced settings does not necessarily indicate that the beds are available for acute care due to the lack of human resources (0.9 mental health practitioners per 100 000) [2] or funds to run the facilities, as was reported for Guinea-Bissau. A worldwide expert consensus process including experts from the African region defined less than 15 beds per 100 000 population as severe shortage and recommended a minimum number of 30 per 100 000 population [43]. Experts from African countries usually argue to increase psychiatric bed numbers [44].

Specialized forensic psychiatric beds and residential facilities for people with mental health problems remain mostly unavailable. The low-income SSA countries did not have specialized forensic beds. The potential lack of a specialized workforce and possible cultural barriers, such as language needs, cultural patterns and beliefs of different African cultures, may affect implementation of forensic services as these cross-cultural issues have mostly been neglected in forensic practice [45]. In some countries (eg, Botswana), while it may be known informally that beds are reserved for forensic patients, the beds are not formally captured by the statistics bureau as such. As is common for LMICs, prison populations with mental health problems often remain untreated [5]. On occasion, staff from hospitals can be asked to assist people with mental health problems in prison, as was reported for Rwanda. In Lesotho, forensic patients were referred to as serving indefinite sentences at ‘Her Majesty’s Pleasure’ to eventually await pardon and release. The process can involve religious and traditional leaders, as well as retired professionals from different fields (ie, legal, scientific, and psychiatric). South Africa was the only country that showed relevant rates of residential housing facilities for persons with a mental health condition at the last data point, but this was still below the global average [2].

The SSA region poses particular challenges for the development of mental health systems, demonstrating that psychiatric reforms recommended by the WHO [2] are not easily transferable from high-income countries to LMICs. Aspects of those reforms, such as bed removals, may have been extrapolated without considering the specific contexts in this group of countries. Per capita mental health expenditures in Africa are the lowest in the world [2], which has implications for the development of mental health systems. A significant proportion of facilities run on church and charitable donations. Furthermore, information is particularly hard to obtain and there is a lack of service and epidemiological data [46]. In Eritrea, there were no data on rates of prison populations available for the entire period of observation, and several countries reported data for one year only. When there were data, registries were often in paper format, thus hindering access and analysis. The fact that several countries reported identical psychiatric bed rates to the WHO for different years of the Atlas Project indicates that the quality of the national registries may be low and that rates may be extrapolated from one year to next without new assessment [47,48].

Although SSA countries had on average lower prison population rates (94 per 100 000 population) compared with global estimates (145 per 100 000 population) and with OECD countries (128 per 100 000 population), there was great heterogeneity between countries. Many countries were marked by civil war and armed conflict, often involving authoritarian and militarized regimes.[49] Mass incarcerations and political detentions often occur under such governments, as seen in Kenya, Nigeria, Uganda, and South Sudan.[49] Rwanda is probably the most prominent example, with an increase in its prison population rate to more than 1800 per 100 000 following the genocide in 1994. In spite of prison population reductions since then, its rate remained over 580 per 100 000, which is currently the second highest in the world [50].

Moreover, African courts often deliver relatively severe sentences with pre-trial and remand prison sentences for minor offences [51]. This leads to high proportions of remanded prison populations. The relationship between income group and incarceration rates seen in this study could indicate that increasing resources were directed into incarcerating more people rather than in improving the quality of imprisonment. In regions moving from war to fragile peace, reforms have taken place aiming to improve access to justice and better confinement conditions, often supported by NGOs such as the Penal Reform International (PRI) [51]. For instance, in Cameroon, a presidential decree recently readjusted sentences across the entire prison population shortening life sentences to 25 years [51]. In Rwanda, following the genocide, restorative justice programs have been put in place to facilitate the reintegration of ex-prisoners [52]. However, penal justice reforms are not a priority for most African governments and international donors [49]. Even though changes of incarceration rates were heterogeneous in SSA, they decreased on average over time as in Central Eastern Europe and Central Asia (CEECA) [19]. Changes of mean incarceration rates over time in SSA were in contrast with strongly increasing rates of imprisonment in Latin America (median increase of 181% per 100 000) [23].

Greater psychiatric bed rates in countries with higher income levels may indicate that the provision of inpatient services was limited by health care budget constraints [53]. Countries in higher income groups also had greater prison population rates, which may indicate that higher incomes could also be a driver of imprisonment in the area. This finding was in line with studies from Latin America [23,54] and the CEECA region [19].

### Strengths and limitations

This is the first study to assess and internationally compare indicators of institutionalization in 46 countries of the SSA region over the period of three decades. Primary data were retrieved from 17 of the 48 countries. Limitations of this study arise from the ecological study design. Incomplete and missing data were further limitations of the study, especially for forensic and residential facilities data which were scarce and did not allow for the description of clear trends. Service data in Africa were often registered in paper formats, which posed particular challenges during the COVID-19 pandemic, as health service planners working from home had limited access to the registries. Finally, our conceptualization of psychiatric facilities was orthodox. While this may be the reality of most of the high-income OECD states, complementary and alternative (traditional and faith-based) mental-health facilities are ubiquitous in many SSA countries and sometimes have more ‘psychiatric beds’ than the orthodox system [55]. For instance, a recent study documented as much as 406 in Kenya, 205 in Ghana, and 82 in Nigeria [55]. Inequitable access to psychiatric care and the uneven geographic distribution of facilities imply to make careful conclusions based on population standardized rates.

### Implications

Data scarcity and lack of access to mental health databases in the SSA region poses a major challenge for service development [46]. Further standardization of data collection methods in SSA countries is required in order to improve comparisons between mental health care systems [56]. At present, no general consensus on minimum psychiatric bed rates has been established for the region, although the normative approach [57] and the observed outcomes approach [58] have been used in other regions.

There has been an overall decreasing trend of psychiatric bed availability in SSA from initially very low rates that may correspond to an increasing crisis in providing acute care for people with severe mental disorders and dual diagnoses, especially when these individuals lack strong family support. In order to develop adequate public policies to address this treatment gap and humanitarian crisis, setting targets and changing trends of psychiatric bed rates are necessary [59]. Psychiatric reform models have been based on experiences from high-income countries and implemented in many LMICs without considering local data, contexts, and the scarcity of resources. Careful assessment of bed rates and trends allows for more tailored planning.

Prison population rates have shown heterogeneous trends and many SSA countries reported substantially greater rates of imprisonment than the global average [50]. On average, these rates are not very different from OECD countries, which implies a tendency to contain people with mental disorders who violate the law within correctional systems rather than the health systems. Further research on prison capacities, types, occupancy, and quality needs to be conducted in the SSA region, in order to improve planning and conditions using a human rights approach [49]. Comparing trends of national data with a group of countries with a similar socio-historic context can be a first step to build more adequate and more effective services.

## CONCLUSIONS

The data presented shows significant issues, in terms of quality of available data and evidence of severe constraints, within both the psychiatric and prison service in SSA. Even at that, there are significant country differences in both quality and reliability of data and assumed availability of both mental health and prison services. Any reformative attempt within the mental health and prison systems, which should be integrated to reflect the cross-cutting nature of need, will do well to be cognizant of these limitations. There is need for more effort at keeping accurate data in the region with which more nuanced understanding of the challenge can be anchored and from which service improvement strategies can be built.

## Additional material


Online Supplementary Document
