# Supplementary material for: Changes in rates of psychiatric beds and prison populations in sub-Saharan Africa from 1990 to 2020
Source: J Glob Health. 2022 Sep 3;12:04054. doi: 10.7189/jogh.12.04054 (PMC9440375; doi:10.7189/jogh.12.04054)
Supplement: Online Supplementary Document [file jogh-12-04054-s001.pdf]

Online Supplement: Changes in rates of psychiatric beds and prison populations in Sub-Saharan Africa from 1990 to 2020

Figure 1: Numbers of specialized forensic psychiatric beds per 100 000 population (1990-2020)

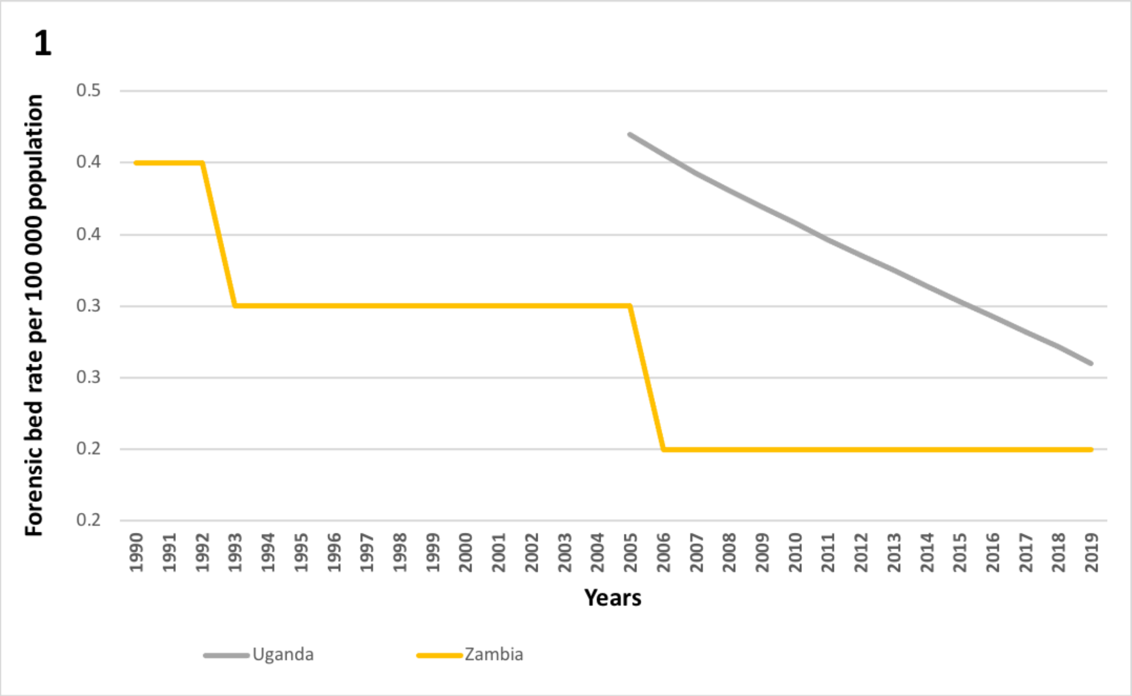

Figure 2: Numbers of residential places for mentally ill people per 100 000 population (1990-2020)

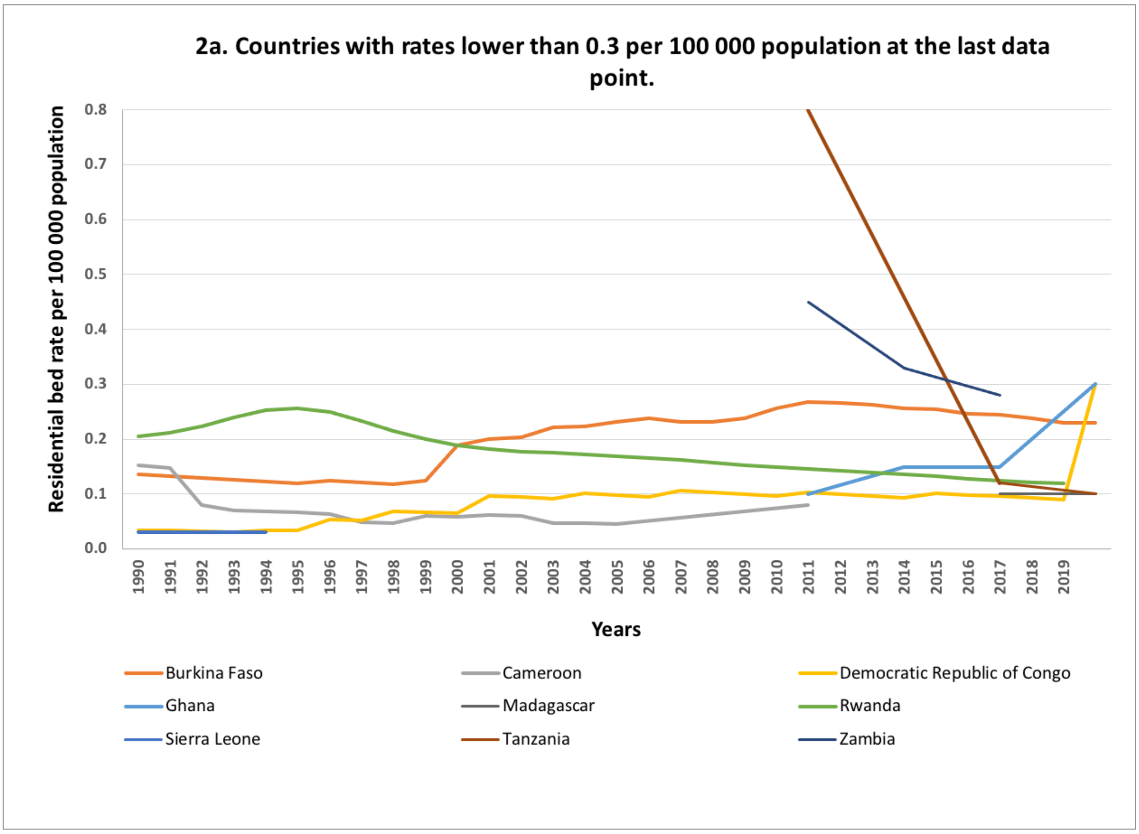

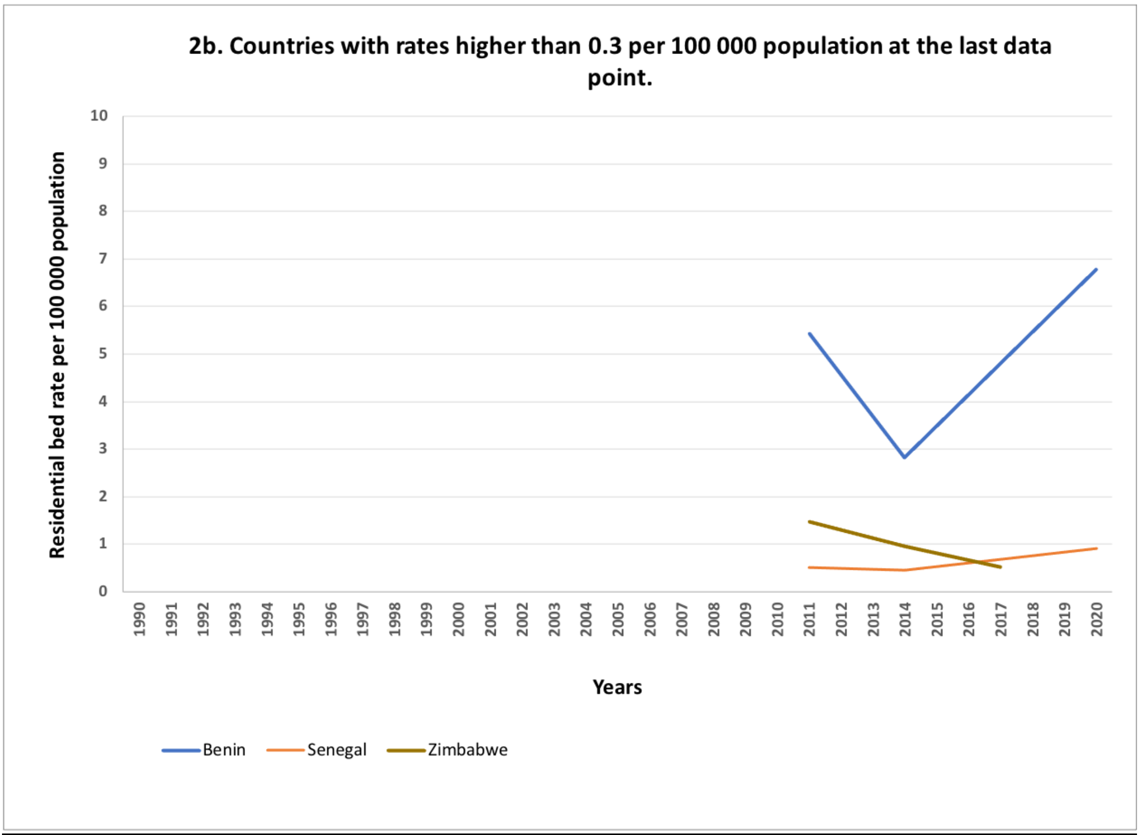
2b. Countries with rates higher than 0.3 per 100 000 population at the last data point.
Residential bed rate per 100 000 population
0
1
2
3
4
5
6
7
8
9
10
1990
1991
1992
1993
1994
1995
1996
1997
1998
1999
2000
2001
2002
2003
2004
2005
2006
2007
2008
2009
2010
2011
2012
2013
2014
2015
2016
2017
2018
2019
2020
Years
Benin
Senegal
Zimbabwe
